# Optimizing the pharmacological component of integrated balance therapy

**DOI:** 10.1016/S1808-8694(15)31116-2

**Published:** 2015-10-20

**Authors:** Maurício Malavasi Ganança, Heloisa Helena Caovilla, Mário Sérgio Lei Munhoz, Cristina Freitas Ganança, Maria Leonor Garcia da Silva, Flavio Serafini, Fernando Freitas Ganança

**Affiliations:** 1 Full Professor of Otorhinolaryngology - Federal University of São Paulo - Brazil - Coordinator of the MS Program in Body Movement Sciences - Universidade Bandeirante de São Paulo; 2 Associate Professor of Neurotology - Federal University of São Paulo; 3 Associate Professor and Head of Neurotology, Federal University of São Paulo - Brazil; 4 Master of Arts in Human Communication Disorders, Federal University of São Paulo - Brazil; 5 MS in Otorhinolaryngology - Federal University of São Paulo; 6 PhD in Otorhinolaryngology - Federal University of São Paulo; 7 Doctor of Sciences, Otorhinolaryngology Head and Neck Surgery, UNIFESP-EPM. Affiliated Professor Responsible for the Department of Vestibular Rehabilitation of the Discipline of Neurotology, UNIFESP-EPM. Responsible for the Discipline of Vestibular Rehabilitation, Mastership in Neurimotor Rehabilitation Sciences, UNIBAN

**Keywords:** meniere's disease/therapy, labyrinth diseases, dizziness, vertigo

## Abstract

**Summary:**

Drug treatment is an important option for the treatment of peripheral vestibular diseases.

**Aim:**

To identify the drug component associated with optimal integrated balance therapy (IBT) for Ménière's disease or other peripheral vestibular disorders.

**Materials and Methods:**

Analysis of a series of patients with Ménière's disease patients or patients with other peripheral vestibular disorders that received IBT involving either no medication or betahistine, cinnarizine, clonazepam, flunarizine or Ginkgo biloba during 120 days.

**Results:**

In Ménière's disease, significant differences were observed for all drug therapies (60 days) versus no medication; betahistine was significantly more effective than all other drugs at 60 and 120 days. For non-Ménière's disorders, significant differences were observed among betahistine, cinnarizine, clonazepam and flunarizine and no medication after 60 days; all drug therapies were significantly more effective than no medication after 120 days; betahistine, cinnarizine or clonazepam were equally effective and betahistine was more effective than flunarizine and EGb 761. All treatment options were well tolerated.

**Conclusions:**

Drug therapies were more effective than no medication in the IBT for patients with Ménière's disease or other peripheral vestibular disorders. Betahistine was the most effective medication for patients with Ménière's disease and was as effective as cinnarizine and clonazepam for other peripheral vestibular disorders.

## INTRODUCTION

According to randomized, double blind, placebo-controlled studies and literature reviews, monotherapeutic strategies may not be enough to bring about complete vertigo resolution.^1^ A precise diagnosis is essential in order to control vertigo^1^, ^2^ The combination of clinical history and otoneurologic findings leads us towards lesion detection and proper diagnosis. Besides its clinical value, otoneurologic assessments may contribute to treatment definition and prognosis, and it also supports patient follow up.^3^

Although there are many options to ameliorate or totally resolve vestibular vertigo and associated symptoms, therapy must be designed based on specific patient disorder, considering the resolution of underlying diseases, vertigo control, neurovegetative and psychoaffective related symptoms, the improvement in vestibular compensation and the prevention of aggravating factors.^1^,^4^ A quick onset of therapeutic action is paramount in order to restore the patient's well being; treatment has to be well tolerated, and bear a low incidence of adverse effects.^1^

Therapeutic results in patients with vestibular vertigo improve significantly with the concurrent use of etiological control, pharmacotherapy, customized vestibular rehabilitation exercises, diet control and life style changes. The use of a combined therapeutic modality may lead to improvements or faster and more long lasting recoveries when compared to monotherapy alone.^1^

Both Nocebo and placebo effects are also present in clinical practice.^4^, ^5^ In a placebo-controlled study of an active drug, vertigo improvement or resolution was seen in only 14.7% of the non-treated patients, and in 40.1% of the control patients who were taking some placebo drug; when only the assumed cause was treated, 36.9% of the patients improved.^6^ Etiologic treatment is Paramount, but it does not offer the patient a significant improvement or vertigo symptoms resolution when used alone.6 In order to promote vestibular compensation, rehabilitation exercises should include habituation of abnormal responses, postural control exercises, visual-vestibular interaction and conditioning activities.^1^,^7^, ^8^ Vestibular rehabilitation exercises were efficient in 51.1% of the patients when used alone.^6^ Malnutrition and bad feeding habits are common worsening factors, and they may even represent likely vertigo etiologic factors.1,8-9 Diet and feeding habit changes improve vertigo in 42.2% of the patients with vestibulopathies.^6^

Many safe and efficient anti-vertigo drugs are currently available. Clinical experience has shown that 16 mg of betahistine TID; 12.5 mg of cinnarizine TID; 0.5 mg of clonazepam, BID; 5 mg of flunarizine, BID; or 80 mg of ginkgo biloba extract (EGb 761), TID may be useful in vertigo control.^3^,^6^,^8^

There is evidence of a significant inverse correlation between the anti-vertigo action of clonazepam, cinnarizine or flunarizine and their daily doses.^10^, ^11^ Betahistine promotes and facilitates central vestibular compensation.^12^, ^13^, ^14^ EGb 761 accelerates postural and locomotor balance and oculomotor function recovery.^15^

Betahistine is an H_3_ heteroreceptor antagonist and an H1 receptor agonist^16^, ^17^ able to improve inner ear microcirculation.^17^ It is used in the treatment of numerous vestibulopathies.^8^,^17^, ^18^, ^19^ There may be side effects such as headaches and epigastric discomfort; gastrointestinal ulcers, asthma and pheochromocytoma are counter-indications to the use of this drug.^8^ H1 receptor blockers and flunarizine, cinnarizine and calcium antagonists inhibit vessel constriction and act as vestibular sedative drugs, used in the treatment of both central and peripheral vertigo.^8^,^20^, ^21^, ^22^, ^23^, ^24^ Fatigue, sleepiness, epigastric discomfort, weight gain, depression and extrapyramidal symptoms are the main adverse effects seen with the use of both drugs. They are both counterindicated in patients with extrapyramidal disorders.^8^ Flunarizine is also employed in the treatment of migraine.^25^ Clonazepam is a benzodiazepine drug that increases the inhibitor effect of gama-amino-butyric acid in the vestibular nuclei, and is useful in vertigo therapy and in controlling anxiety and panic spells in vertiginous patients. Patients may experience sleepiness, fatigue and drug addiction. Myasthenia gravis and narrow angle acute glaucoma are its counterindication.^8^, ^9^, ^10^, ^11^ EGb 761 bears hemodynamic, hemorheologic, metabolic and neural effects.^8^,^26^ It is used to treat both central and peripheral vertigo.^8^,^27^, ^28^ Headache, hypotension and gastrointestinal disorders are its main side effects.8

Vertigo improvement was seen with drug therapy alone in 75.1% of the patients with peripheral vestibulopathies and in 39.8% of the patients with central vestibular disorders.^29^

Results attained from a single treatment modality were usually worse than those attained with some combined therapy. Nonetheless, a combination of therapy modalities brought about vertigo improvement in 96.0% of the cases.^1^,^6^,^8^

The goal of the present investigation is to assess the vertigo integrated therapy (VIT) efficacy and safety, based on concurrent approaches that include etiologic treatment, customized rehabilitation exercises, diet control and life style changes, with or without medication

## PATIENTS AND METHODS

This study was approved by the Ethics Committee of our institution, under protocol # 0973/04.

We consulted the charts of 1,100 outpatients with established Ménière's disease or other peripheral vestibulopathies treated by VIT, including etiologic treatment, personalized rehabilitation exercises, diet control and life style changes, with or without antivertigo substance use, and check if there was vertigo improvement. Established Ménière's disease was defined based on the criteria from the American Academy of Otorhinolaryngology-Head and Neck Surgery: 1) Two or more spontaneous vertigo episodes lasting 20 minutes or more; 2) hearing loss documented by audiometry in at least one occasion; 3) tinnitus or ear fullness in the treated ear; 4) ruling out other causes.^30^ Ménière patients who suffered at least three vertigo episodes in the two previous months were considered eligible. Patients with peripheral vestibulopathies that were not Ménière's disease were included if they suffered recurrent vertigo, instability between crises or continuous diziness in the two prior months.^8^ All the patients received treatment for 120 consecutive days.

The patients underwent a careful examination, including clinical history; ear, nose and throat exam; audiometry and vestibular assessment, including electronystagmography before and after VIT. Auditory assessment was based on threshold tonal audiometry, speech recognition threshold and immitance test. We included tests of brain stem auditory response and/or electrocochleography when necessary. Balance assessment included caloric, gait and posture tests, positional nystagmus, spontaneous nystagmus, semi-spontaneous nystagmus, saccadic movements, pendular tracking, optokinetic nystagmus and self-rotation of the head.

After detecting the assumed etiology for the vestibular disorder, we started a specific treatment for the underlying disease. A personalized rehabilitation program, including habituation, sensorial substitution or adaptative mechanisms were applied according to the specific vestibular disorder suffered by each patient.

The patients were instructed to eat lavishly during breakfast, have a light lunch and an even lighter dinner, avoiding intervals greater than 3 hours in between meals, as well as avoiding the use of refined sugar, coffee, alcohol and tobacco. They were encouraged to practice physical exercises according to their own physical condition.

The patients were randomized to receive no medication or oral drug therapy made up of 16 mg of betahistine TID; 12.5 mg of cinnarizine TID; 80 mg of EGb 761, TID; 0.5 mg of clonazepam BID; or 0.5 mg of flunarizine, once a day before going to sleep. The use of other antivertiginous drug was not allowed.

The patients were examined in three occasions: at study admittance; two and four months after therapy onset. The variable used to assess treatment efficacy was the patient's overall impression. Efficacy was assessed according to the patient's subjective response after the end of each treatment period. Efficacy assessment used the following classifications: 1 = no symptoms (full recovery), 2 = very good improvement, 3 = good improvement, 4 = mild response and 5 = no response. The patients who reported having very good, good and mild improvement were included in the category of those who improved (with partial improvement). Tolerability was assessed by the investigators and the patients themselves at the end of each treatment period.

### Statistical analysis

We used statistical analysis in order to detect possible differences in the efficacy rates between patients assigned to the six VIT groups, considering the disease presented and treatment response. Treatment groups were compared using the chi-squared Pearson's test at a 5% significance level.

## RESULTS

Of the 1,100 patients selected for the study, 603 (54.8%) were women and 497 (45.2%) were men; average age was of approximately 48 years; 283 (25.%) had Ménière's disease and 817 (74.3%) had peripheral vestibular disorders. Table 1 depicts patient distribution as to having Ménière's disease or other peripheral vestibular disorders according to VIT treatment group. The rate of Ménière patients or those with other vestibular disorders was not different among the groups (p=0.105). Table 2 shows the treatment effects on Ménière's disease patients.

**Table 1:** Patient distribution according to Vertigo Integrated Therapy and the vestibular disorder.

VIT Group	Ménière's Disease		Other vestibular disorders		Total
	N	%	N	%	
Betahistine	62	32.1	131	67.9	193
Cinnarizine	60	29.7	142	70.3	202
Clonazepam	44	23.5	143	76.5	187
Flunarizina	41	22.9	138	77.1	179
EGb 761	41	23.3	135	76.7	176
No medication	35	21.5	128	78.5	163
Total	283	25.7	817	74.3	1,100

**Table 2:** Results from the Vertigo Integrated Therapy in patients with Ménière's disease.

VIT Group	Asymptomatic				Improved				No change			
	60 days		120 days		60 days		120 days		60 days		120 days	
	N	%	N	%	N	%	N	%	N	%	N	%
Betahistine	6	9.7	31	50.0	45	72.6	24	38.7	11	17.7	7	11.3
Cinnarizine	4	6.7	17	28.3	36	60.0	28	46.7	20	33.3	15	25.0
Clonazepam	3	6.8	11	25.0	24	54.5	20	45.5	17	38.6	13	29.5
Flunarizine	2	4.9	8	19.5	18	43.9	19	46.3	21	51.2	14	34.1
EGb 761	2	4.9	8	19.5	17	41.5	18	43.9	22	53.7	15	36.6
No medication	0	0.0	5	14.3	5	14.3	14	14.0	30	85.7	16	45.7

As to the percentage of asymptomatic patients with Ménière's disease and those with it that improved after 60 days of treatment, statistically significant differences were seen between betahistine (p<0.0001), cinnarizine (p<0.0001), clonazepam (p<0.0001), flunarizine (p=0.0014) or EGb 761 (p=0.0027) and VIT without medication. The percentage of patients who presented full and partial resolution was greater in all the groups that received VIT with medication when compared to those who did not. The group treated with betahistine presented a significantly higher full and partial resolution when compared to cinnarizine (p=0.048), clonazepam (p=0.016), flunarizine (p<0.0005) or EGb 761 (p=0.0001). The group treated with cinnarizine had a higher partial and full resolution rate when compared to the group receiving EGb 761 (p=0.042).

As to the percentage of patients with Ménière's disease who were asymptomatic or improved after 120 days of treatment, statistically significant differences were observed favoring betahistine (p<0.001) and cinnarizine (p=0.038) when compared to no medication. Patients treated with betahistine presented a significantly higher percentage of full and partial resolution when compared to those under cinnarizine (p=0.049), clonazepam (p=0.018), flunarizine (p=0.005) or EGb 761 (p=0.002).

Table 3 depicts treatment effects in patients with vestibular disorders, excluding Ménière's disease.

**Table 3:** Vertigo Integrated Therapy results in patients with vestibulopathies, excluding Ménière's disease.

VIT Group	Asymptomatic				Improved				Unaltered			
	60 days		120 days		60 days		120 days		60 days		120 days	
	N	%	N	%	N	%	N	%	N	%	N	%
Betahistine	14	10.7	71	54.2	91	69.5	49	37.4	26	19.8	11	8.4
Cinnarizine	10	7.0	48	33.8	92	64.8	69	48.6	40	28.2	25	17.6
Clonazepam	11	7.7	48	33.6	97	67.8	75	52.4	35	24.5	20	14.0
Flunarizine	8	5.8	49	35.5	80	58.0	70	50.7	50	36.2	19	13.8
EGb 761	6	4.4	40	29.6	74	54.8	70	51.9	55	40.7	25	18.5
No medication	2	1.6	17	13.3	63	49.2	49	38.3	63	49.2	62	48.3

As to the percentage of patients with peripheral vestibular disorders, excluding Mèniére, who were asymptomatic and improved after 60 days of treatment, statistically significant differences were observed between betahistine (p<0.0001), cinnarizine (p<0.0005), clonazepam (p<0.0001) or flunarizine (p=0.0322) and no medication. The groups treated with betahistine, cinnarizine, clonazepam or flunarizine presented higher percentages of complete and partial improvements when compared to the groups that did not receive medication treatment. Betahistine was associated to a higher rate of partial and complete symptoms relief when compared to flunarizine (p=0.0029) or EGb 761 (p=0.002). Cinnarizine was associated with a higher percentage of complete and partial symptoms relief when compared to EGb 761 (p=0.,0276). Clonazepam was associated to a higher rate of complete and partial symptoms relief when compared to flunarizine (p=0.0320) or EGb 761 (p=0.0038).

As to the percentage of patients with other peripheral vestibular disorders, Ménière excluded, who were asymptomatic and improved after 120 days of treatment, statistically significant differences were observed between betahistine (p<0.001), cinnarizine (p<0.001), clonazepam (p<0.001), flunarizine (p=0.001) or EGb 761 (p=0.001) and no medication. Betahistine, cinnarizine, clonazepam, flunarizine and EGb 761 were associated to a higher rate of complete and partial improvements when compared to no medication. Betahistine was associated to a higher rate of complete and partial improvement when compared to flunarizine (p=0.025) or EGb 761 (p=0.001).

All treatment modalities were well tolerated, with a low incidence of adverse effects. Severe adverse effects were not reported. Medication termination was not required in any of the cases. Table 4 depicts adverse effect events in the groups that received VIT. When compared with ciniarizine, clonazepam and flunarizine, betahistine presented a substantially lower incidence of the most seen adverse effects such as sleepiness and depression, and a comparable or lower incidence of most of the other adverse effects when compared to the group that did not receive any medication.

**Table 4:** Adverse events prevalence in the groups that received the Vertigo Integrated Therapy.

Adverse Event	VIT Group													
	Betahistine		Cinnarizine		Clonazepam		Flunarizine		EGb 761		No medication		Total	
	N	%	N	%	N	%	N	%	N	%	N	%	N	%
Sleepiness	0	0.0	48	23.8	49	26.2	53	29.6	0	0.0	0	0.0	150	13.3
Depression	4	2.1	15	7.4	17	9.1	24	13.4	5	2.8	28	17.1	93	8.5
Headache	16	8.3	15	7.4	11	5.9	17	9.5	16	9.1	13	8.0	88	8.0
Anxiety	6	3.′	5	2.5	0	0.0	4	2.2	8	4.5	24	14.7	47	4.3
Epigastric discomfort	10	5.2	6	2.9	5	2.7	5	2.8	11	6.3	7	4.3	44	4.0
Urinary retention	3	1.6	12	5.9	4	2.1	17	9.5	2	1.1	3	1.8	41	3.7
Weight gain	2	1.0	15	7.4	2	1.1	18	10.1	1	0.6	2	1.2	39	3.5
Dry mouth	0	0.0	19	9.4	0	0.0	17	9.5	0	0.0	0	0.0	36	3.3
Fatigue	2	1.0	9	5.5	4	2.1	11	6.1	3	1.7	6	3.7	3.5	3.2
Insomnia	4	2.1	4	2.5	2	1.1	3	1.7	3	1.7	11	6.7	27	2.5
Libido reduction	1	0.5	5	2.5	5	2.7	6	3.4	2	1.1	2	1.2	21	1.9
Palpitation	3	1.6	4	2.0	3	1.6	5	2.8	3	1.7	3	1.8	13	1.2
Nausea	2	1.0	2	1.0	1	0.5	2	1.1	3	1.7	2	1.2	12	1.1
Dyspepsia	2	1.0	2	1.0	1	0.5	1	0.6	3	1.7	3	1.8	12	1.1
Lipothymia	1	1.0	2	1.0	0	0.0	1	0.6	1	0.6	3	1.8	8	0.7
Arterial hypotension	1	1.0	2	1.0	0	0.0	2	1.1	1	0.6	1	0.6	7	0.6
Blurred vision	0	0.0	0	0.0	1	0.5	3	1.7	0	0.0	1	0.6	5	0.4
Skin rash	0	0.0	0	0.0	0	0.0	0	0.0	0	0.0	1	0.6	1	0.1

## DISCUSSION

The present study provides enough evidence of the antivertiginous efficacy and excellent tolerability of a VIT that included etiologic treatment, personalized rehabilitation exercises, diet control, life style changes and drug therapy with betahistine, cinnarizine, clonazepam, flunarizine or EGb 761.

It has been established that betahistine^8^,^17^, ^18^, ^19^, cinnarizine^23^, ^24^, clonazepam^8^,^11^, flunarizine^23^, ^24^, or EGb 761^8^,^27^, ^28^ ameliorate vestibular vertigo. In the treatment of Ménière's diseases, betahistine proved eficatious^8^, ^31^ and is more efficient than cinnarizine in reducing instability after vestibular neurectomy and in increasing vestibular compensation efficiency.^32^ Flunarizine also has a positive therapeutic effect.^33^

In our study, all five substances studied were more efficient when compared to no medication at all after two months of VIT in patients with Ménière's disease. After four months of therapy, only betahistine or cinnarizine were more efficient than no medication. Betahistine was more efficient than cinnarizine, clonazepam, flunarizine or EGb 761 after two and four months of therapy. Betahistine, cinnarizine or clonazepam have all a better antivertiginous effect significantly earlier than that of flunarizine, EGb 761 or no medication.

In treating peripheral vestibular vertigo, betahistine^8^,^18^, ^19^, cinnarizine^6^,^8^,^23^,^29^, clonazepam^6^,^8^,^11^,^29^, flunarizine^6^,^8^,^11^,^29^, and EGb 761^6^,^8^,^11^,^29^ proved to be efficacious; on the other hand, betahistine proved to be more efficient than flunarizine^34^ and similar to EGb 761.^28^

After two and four months of VIT in patients with peripheral vestibular disorders, excluding Ménière's disease, betahistine, cinnarizine, clonazepam or flunarizine were more efficient than no medication at all. All the drugs studied reached their best antivertiginous effects after four months of treatment.

It is possible that some of the therapeutic failures have happened because of the very difficulty in detecting or controlling an underlying disease, the patient's difficulty in complying with treatment protocol, perform the rehabilitation exercises, follow diet recommendations and change life style habits. On the other hand, etiologic control of aggravating factors and the responses from the balance rehabilitation program may have varied according to the patient and the vestibular disorder, and may have had a different impact on the groups that underwent drug therapy.

Patient compliance is important in order to reach the therapeutic effect desired, and the incidence of adverse effects may impact this compliance. Therefore, it is worth mentioning that, in the present study, betahistine was associated to a substantially lower incidence of adverse effects such as sleepiness and depression when compared to cinnarizine, clonazepam and flunarizine, and a comparable or lower incidence of most of the other adverse events when compared to the group that did not receive any medication.

The highest rates of improvement seen with a VIT that included medication usage were possible due to the summation of favorable effects reached by combined strategies. Favorable results associated to a good tolerability suggest that VIT is a good option to use in order to control vertigo in patients with Ménière's disease or other peripheral vestibular disorders.

## CONCLUSION

In a combination of therapeutic modalities used to treat Ménière's disease or other peripheral vestibular disorders, betahistine, cinnarizine, clonazepam, flunarizine or EGb 761 are more efficient than not using medication at all; betahistine is the most efficient medication to be used in patients with Ménière's disease; betahistine, cinnarizine or clonazepam are equally efficient, and betahistine is more efficient than flunarizine or EGb 761 in patients with peripheral vestibular disorders, not considering Ménière's disease.

## ACKNOWLEDGMENTS

The authors wish to thank the Clinical Research Institute of São Paulo and Spectrum Consultoria e Projetos em Saúde S/C Ltda. For the statistical analysis of the present study.

## References

[1] Ganança MM, Caovilla HH, CF Claussen, CT Haid, B Hofferberth (1999). Equilibrium research and equilibriometry in modern treatment..

[2] Bhansali SA, JA Goebel (2001). Practical management of the dizzy patient..

[3] Hain TC, Uddin M (2003). Pharmacological treatment of vertigo.. CNS Drugs.

[4] Macedo A, Farre M, Banos JE (2003). Placebo effect and placebos: what are talking about?. Some conceptual and historical considerations. Eur J Clin Pharmacol.

[5] Ferreres J, Banos JE, Farre M (2004). Nocebo effect: the other side of placebo.. Med Clin (Barc).

[6] Caovilla HH, Ganança MM, A Cesarani, D Alpini (1990). Diagnosi e trattamento dei disturbi delléquilibrio nelletà evolutiva ed involutiva..

[7] SJ Herdman (2000).

[8] Ganança MM, Munhoz MSL, Caovilla HH Silva (2006). MLG. Managing vertigo..

[9] Ganança MM, Ramos S, Ramos RF, Mangabeira Albernaz PL, Caovilla HH, CF Claussen, MV Kirtane, K Schlitter (1988). Vertigo, nausea, tinnitus and hypoacusia in metabolic disorders..

[10] Mangabeira Albernaz PL (1988). Calcium antagonists as peripherally acting labyrinthine suppressants in humans.. Acta Otolaryngol Suppl.

[11] Ganança MM, Caovilla HH, Ganança FF, Ganança CF, Munhoz MSL, Silva MLG, Serafini F (2002). Clonazepam in pharmacological treatment of vertigo and tinnitus.. Int Tinnitus J..

[12] Dutia MB (2000). Betahistine, vestibular function and compensation: in vitro studies of vestibular function and plasticity.. Acta Otolaryngol Suppl.

[13] Lacour M, Tighilet B (2000). Vestibular compensation in the cat: the role of the histaminergic system.. Acta Otolaryngol Suppl.

[14] Lacour M, Sterkers O (2001). Histamine and betahistine in the treatment of vertigo: elucidadtion of mechanisms of action.. CNS Drugs..

[15] Ez-Zaher L, Lacour M, CF Claussen, MV Kirtane, K Schlitter (1988). Vertigo, nausea, tinnitus and hypoacusia in metabolic disorders..

[16] Arrang JM, Garbarg M, Quach TT, Dam Trung Tuong M, Yeramian E, Schwartz JC (1985). Actions of betahistine at histamine receptors in the brain.. Eur J Pharmacol.

[17] Dziadziola JK, Laurikainen EL, Rachel JD, Quirk WS (1999). Betahistine increases vestibular blood flow.. Otolaryngol Head Neck Surg.

[18] Tran Ba Huy P, Meyrand MF (1992). Le dichlorhydrate de betahistine en 2 prises ou en 3 prises par jour.. J Fr Oto-Rhino-Laryngol.

[19] Mira E, Guidetti G, Ghilardi PL, Fattori B, Malaninno N, Maiolino L, Mora R, Ottoboni S, Pagnini P, Leprini M, Pallestrini E, Passali D, Nuti D, Russolo M, Tirelli G, Simoncelli C, Brizi S, Vicini C, Frasconi P (2003). Betahistine dihydrochloride in the treatment of peripheral vestibular vertigo.. Eur Arch Otorhinolaryngol.

[20] Godfraind T, Towse G, van Nueten JM (1982). Cinnarizine - a selective calcium entry blocker.. Drugs Today..

[21] Norris CH (1988). Drugs affecting the inner ear: a review of their clinical efficacy, mechanisms of action, toxicity and place in therapy.. Drugs.

[22] Oosterveld WJ, Hofferberth B (1988). Ca++ and the vestibular system.. Acta Otolaryng Suppl (Stockh).

[23] Ganança MM (1990). How to use cinnarizine and flunarizine in the treatment of vertigo.. Proc. Int. Symposium for prof. G. Portmanns Centenary, Ankara, Turkey.

[24] Mangabeira Albernaz PL (1978). Ganança MM, Novo NF, Paiva ER.. Flunarizine and cinnarizine as vestibular depressants: a statistical study. ORL (Basel).

[25] Greenberg DA (1986). Calcium channel antagonists and the treatment of migraine.. Clin Neuropharmacol.

[26] Clostre F (1999). Ginkgo biloba extract (EGb761): state of knowledge in the dawn of the year 2000.. Ann Pharm Fr.

[27] Ganança MM, Caovilla HH, Ganança FF, Serafini F, A Cesarani, D Alpini (1994). Equilibrium disorders: brainstem and cerebellar pathology..

[28] Cesarani A, Meloni F, Alpini D, Barozzi S, Verderio L, Boscani PF (1998). Ginkgo biloba (EGb 761) in the treatment of equilibrium disorders.. Adv Ther.

[29] Ganança MM, Mangabeira Albernaz PL, Caovilla HH, Ganança FF, CF Claussen, MV Kirtane, D Schneider (1994). Vertigo, hypoacusia and tinnitus due to central disequilibrium..

[30] (1995). Committee on Hearing and Equilibrium guidelines for the diagnosis and evaluation of therapy in Ménière's disease.. American Academy of Otolaryngology-Head and Neck Foundation, Inc. Otolaryngol Head Neck Surg..

[31] Claes J, Van de Heyning PH (2000). A review of medical treatment for Ménière's disease.. Acta Otolaryngol Suppl..

[32] Colletti V (2000). Medical treatment in Ménière's disease: avoiding vestibular neurectomy and facilitating postoperative compensation.. Acta Otolaryngol Suppl.

[33] Haid T (1988). Evaluation of flunarizine in patients with Ménière's disease.. Subjective and vestibular findings. Acta Otolaryngol Suppl.

[34] Fraysse B, Bebear JP, Dubreuil C, Berges C, Dauman R (1991). Betahistine dihydrochloride versus flunarizine.. A double-blind study on recurrent vertigo with or without cochlear syndrome typical of Ménière's disease. Acta Otolaryngol Suppl.

